# Decellularized lymph node scaffolds accelerate restoration of lymphatic drainage in rat hind limb lymphedema

**DOI:** 10.1002/btm2.70056

**Published:** 2025-07-31

**Authors:** Yang Jian, Jian Zhou, Wenjie Pan, Jiayin Chen, Yanji Zhang, Yanqi Li, Xin Liu, Shune Xiao, Chenliang Deng, Zairong Wei

**Affiliations:** ^1^ Department of Burns and Plastic Surgery Affiliated Hospital of Zunyi Medical University Zunyi Guizhou China; ^2^ The Collaborative Innovation Center of Tissue Damage Repair and Regeneration Medicine of Zunyi Medical University Zunyi Guizhou China

**Keywords:** artificial lymphoid tissue, decellularized lymph node, immediate lymphatic reconstruction, lymphatic vessels, lymphedema

## Abstract

**Background:**

There is a lack of effective lymphedema prevention methods. The objective of this study was to investigate the ability of decellularized lymph nodes (dLNs) transplantation to prevent hindlimb lymphedema.

**Methods:**

Porcine dLNs were prepared using 1% sodium dodecyl sulfate and 1% Triton X‐100, and the effectiveness of decellularization was assessed by histological assessment and DNA quantification. Lymph node (LN) fragments and dLNs were transplanted into mice, and samples were collected for evaluating biocompatibility at the fourth week postsurgery. Thirty‐six SD rats were separated into a control group (lymphatic dissection), a dLNs group (lymphatic dissection and dLNs transplant) and a sham group (inguinal skin circumferentially incised). Hindlimb circumference was monitored every 3 days. Indocyanine green lymphography was performed before and every week after surgery. Samples were collected for histological assessment at the second and fourth weeks.

**Results:**

The dLNs showed virtually complete absence of cellular material, maintenance of spatial structures, and good biocompatibility and induced immune cell infiltration. Compared with that of the control group, the average hindlimb circumference of the dLN group was significantly reduced on postoperative days (PODs) 8, 12, and 16, and that of the sham group was significantly reduced on PODs 4, 8, 12, 16, and 20. The sham group exhibited intact inguinal LNs and lymphatic drainage. Neonatal lymphatic vessels (LVs) were observed in the dLN group, and obvious dermal backflow was observed in the control group. Transplanted dLNs induced the infiltration of immune cells, which subsequently integrated into the preexisting lymphatic system. Compared with those in the control group or sham group, the number of LYVE‐1^+^ LVs in the affected limb was greater in the dLN group.

**Conclusion:**

The dLNs scaffolds induced the infiltration of immune cells and promoted LVs regeneration, which integrated into the preexisting lymphatic system to accelerate the restoration of lymphatic drainage.

**Translational Impact Statement**

This study demonstrates that decellularized lymph nodes (dLNs) scaffolds exhibit excellent biocompatibility and effectively promote lymphatic vessels regeneration and integration into the host lymphatic system. The findings suggest that dLNs transplantation could serve as a promising preventive or therapeutic strategy for lymphedema. In the future, strategies based on dLNs scaffolds may accelerate lymphatic drainage recovery and induce immune cells infiltration, offering a new approach for lymphatic structure regeneration and regenerative medicine.

## 1 INTRODUCTION

Lymphedema is a chronic progressive disease that clinically manifests as fibrosis, limb swelling, cellulitis, skin lesions, limb dysfunction, and low quality of life^1^ and affects approximately 250 million people worldwide.^2^ In developed countries and in China, cancer‐related lymphedema (CRL) is the most common form.^3^, ^4^ Lymphatic dissection and regional nodal irradiation are the main underlying causes.^5^ Due to a lack of effective treatments, prevention is crucial for lymphedema and its complications from occurring.

Various attempts have been made to prevent the development and progression of lymphedema. Clinical practice guidelines recommend several means to prevent lymphedema, including prospective surveillance programs (PSP), prophylactic compression sleeves (PCS), sentinel lymph node (LN) biopsy, and axillary reverse mapping (ARM).^6^ However, these methods require specialized personnel, good patient compliance, specific indications, and the oncologic safety (such as ARM), which limit their promotion and application and have uncertain preventive effects.^6^, ^7^ Therefore, novel prevention strategies need to be developed.

Lymphatic system injury is an initiating factor for CRL occurrence.^8^ It causes lymph to pool in tissue spaces, chronic inflammation with Th1/Th2 cell imbalance, and adipose tissue deposition and fibrosis, which further impair the lymphatic system.^9^ Therefore, some methods to reconstruct the lymphatic system have been developed to prevent CRL. Immediate lymphatic reconstruction (ILR) is a procedure where lymphaticovenous anastomosis is performed under a microscope. It is used for CRL prevention in patients who have undergone LN dissection. Some short‐term, non‐randomized controlled studies have proven its effectiveness.^6^, ^10^, ^11^ Most importantly, the oncological safety of ILR remains controversial. Vascularized LN transfer (VLNT) is another approach for reconstructing lymphatic drainage; VLNT combined with immediate breast reconstruction has been used for CRL prevention.^12^ Moreover, VLNT has been confirmed to promote the regeneration of lymphatic vessels (LVs) and recover immune function locally and systemically.^13^ This approach seems capable of achieving complete reconstruction of the lymphatic system. However, VLNT may lead to donor‐site lymphedema and the limited donor sites.^14^, ^15^ Moreover, ILR and VLNT require professional personnel and superior microsurgical techniques,^6^, ^10^, ^11^ which limit their promotion and application. Therefore, replacing VLNT with tissue‐engineered approaches seems to be a promising method for CRL treatment or prevention.^16^

Artificial LNs (aLNs) have been proven to induce immune cell colonization and LVs regeneration.^17^, ^18^, ^19^ Suematsu et al.^20^ fabricated artificial lymphoid tissues from mouse thymus‐derived stromal cells and bovine collagen, which can induce the formation of lymph node‐like structures and immune responses. However, it cannot fully replicate the native LN structure with afferent and efferent LVs. Lenti et al.^21^ used decellularized splenic stromal and LN stromal cells to induce aLN. After transplantation to lymphadenectomized sites, aLN integrated into the endogenous LVs and efficiently restored lymphatic drainage and perfusion. It is therefore potentially valuable to reconstruct the lymphatic drainage system by preparing aLN to prevent or treat lymphedema.^22^, ^23^, ^24^ Nevertheless, there are currently no robust engineered LNs capable of replicating the functions of native LNs due to the complex cellular composition and microarchitecture.^25^ To replicate the complex structure, Cuzzone et al.^26^ used decellularized LNs (dLNs) of mice as template scaffolds to engineer mouse LN. Results indicated that the original tissue structure was retained after decellularization and dLNs successfully delivered white blood cells throughout the body. Subsequently, several studies further confirmed that dLNs can induce immune cell infiltration and maintain immune function.^27^, ^28^, ^29^ Whether dLNs contribute to the recovery of immune function and LVs regeneration for lymphedema prevention in preclinical models of LN resection remains unknown.

Here, we prepared porcine dLNs using 1% sodium dodecyl sulfate (SDS) combined with 1% Triton X‐100 and showed that dLNs transplantation at the site of resected LN promotes LVs regeneration and may restore immune function. Upon transplantation, dLNs were integrated into the endogenous LVs, reconnecting the disrupted lymphatic system and effectively accelerating lymphatic drainage and perfusion. Moreover, T cells and B cells infiltration was induced after dLNs transplantation, with the infiltrated immune cells showing a distribution resembling that in native LN. This suggests that dLNs may be involved in immune function regulation. These findings provide preliminary preclinical evidence for dLNs in preventing lymphedema and serving as scaffolds for aLNs.

## 2 METHODS

### 2.1 Decellularisation of porcine LNs

The neck LNs of three locally market‐purchased pigs from Zunyi (aged 1–2 years, 100–150 kg, no gender restrictions) were preserved at −80°C. The LNs were cut into fragments (approximately 0.3 cm^3^); soaked and oscillated in ultrapure water for 16 h (10 mL/g tissue, 180 rpm); decellularized successively by oscillation in a solution of 1% sodium dodecyl sulfate (SDS) (Meilunbio, Da Lian, China) for 18 h and a solution of 1% Triton X‐100 (Abcbio, Xi An, China) for 1 h (10 mL/g tissue, 180 rpm); soaked and oscillated in ultrapure water for another 24 h; soaked and oscillated in a solution of 1% (v/v) penicillin–streptomycin for 2 h (10 mL/g tissue); and soaked and oscillated in phosphate‐buffered saline (PBS) solution for 2 h (10 mL/g tissue). The freeze‐drying process was performed using a freeze drier (Telstar, Barcelona, Spain) at −80°C and 0.000 mbar for 28 h. Afterwards, the product was sealed, sterilized with ethylene oxide, and stored at 4°C.

### 2.2 Characterization of dLNs

#### 2.2.1 DNA quantification

DNA quantification of dLNs and normal LNs (three samples/group) was performed using a TaKaRa MiniBEST Universal Genomic DNA Extraction Kit Ver. 5.0 following the manufacturer's instructions (Servicebio, Wu Han, China). In brief, samples were weighed and then digested with 180 μL Buffer GL, 20 μL proteinase K, and 10 μL RNase A (10 mg/mL) at 56°C for 2 h. After the samples were lysed, 200 μL Buffer GB and 200 μL absolute ethyl alcohol were added. Then the mixture was centrifuged at 12,000 rpm for 2 min. The filtrate was discarded. Next, 500 μL Buffer WA was added to the resulting solution, followed by centrifuging at 12,000 rpm for 1 min. The filtrate was discarded. After that, 700 μL Buffer WB was added, and the solution was centrifuged at 12,000 rpm for 1 min. The filtrate was discarded again. This step of adding Buffer WB and centrifuging was repeated once more, followed by centrifuging at 12,000 rpm for 2 min. Then 50–200 μL Elution Buffer was added to the final solution, which stood at room temperature for 5 min. Finally, the solution was centrifuged at 12,000 rpm for 2 min to elute DNA, and spectrophotometric analysis was performed on the purified DNA.

### 2.3 Microstructure of dLNs scaffolds

The microstructure of the dLNs and fragments of normal LN (three samples/group) was investigated using a scanning electron microscope (SEM). The samples were washed with PBS solution for three times (5 min each time), then fixed in 2.5% glutaraldehyde solution for 2 h at room temperature, and washed twice again with PBS solution (5 min each time). Then sequentially dehydrate in 50%, 70%, 80%, 90%, 95%, 70%, 80%, 90%, 95%, and 100% alcohol for 15 min respectively, then dried using a critical point dryer. After being coated with gold–palladium in a sputter coater and mounted on metal stubs, the specimens were then observed with an SEM (Hitachi, Tokyo, Japan) at an electron‐accelerating voltage of 15 kV.

### 2.4 Subcutaneous dLNs implantation

**FIGURE 1:**
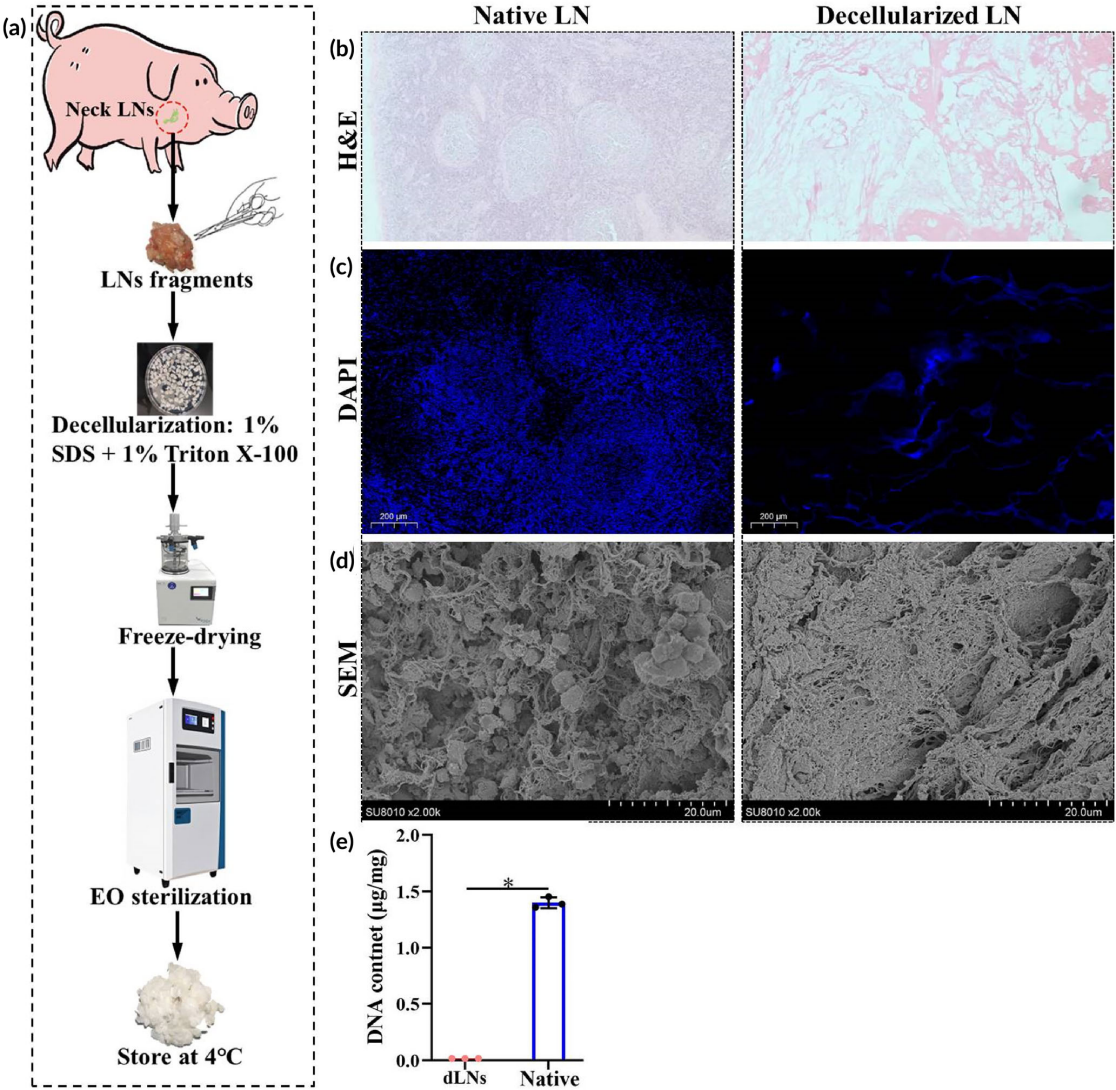
Preparation and characterization of dLNs. (a) Diagram of the decellularized process. (b) HE staining of native LNs (left) and dLNs (right). Scale bar = 500 μm for HE staining. (c) DAPI staining results for native LNs (left) and dLNs (right). Scale bar = 20 μm. (d) SEM images of native LNs (left) and dLNs (right), scale bar = 20 μm for SEM. (e) DNA quantification results for LNs and dLNs (*n* = 3/group, * indicated *p* < 0.05, student's *t*‐test). LNs, lymph nodes; SDS, sodium dodecyl sulfate; EO, ethylene oxide; H&E, hematoxylin and eosin; DAPI, 4′,6‐diamidino‐2‐phenylindole dihydrochloride; SEM, scanning electron microscopy.

To detect the biocompatibility of dLNs and determine whether dLNs could be repopulated with immune cells, frozen porcine LNs fragments and dLNs were transplanted into the subcutaneous regions of the backs of the mice. Briefly, a 0.5 cm full‐thickness incision was made on the left and right sides of the backs of five mice, and LNs fragments and dLNs were transplanted subcutaneously (Figure 1a). Then, the skin was closed. The animals were subsequently sacrificed 4 weeks later, and the LNs fragments and dLNs were excised en bloc for histological examination.

### 2.5 Animals and treatments

Lymphedema is more common in females,^7^ so to align with clinical situations, only female animals were used in our study. Five female mice (C57BL/6, 6–8 weeks old) and thirty‐six female Sprague Dawley (SD) rats (6–8 weeks old, 250–280 g) were obtained from Enswell Biotechnology Co. (Chongqing, China). The animals were placed in controlled environments (12‐h light/dark cycle; 20°C–26°C; 40%–60% humidity) and had free access to bacteria‐free water and food. This work has been reported in accordance with the ARRIVE guidelines (Animals in Research: Reporting in Vivo Experiments).^30^ After 1 week of adaptive feeding, the experiment was conducted. The animal experiments were performed under inhalation anesthesia using 2% isoflurane and were conducted following protocols approved by the Medical University of Zunyi Animal Care and Use Committee (zyfy‐an‐2023‐0220).

To detect the biocompatibility of dLNs and determine whether dLNs could be repopulated with immune cells, frozen porcine LNs fragments and dLNs were transplanted into the subcutaneous regions of the backs of five female mice. Briefly, a 0.5 cm full‐thickness incision was made on the left and right sides of the backs of five mice, and LN fragments and dLNs were transplanted subcutaneously (Figure 1a). Then, the skin was closed. The animals were subsequently sacrificed 4 weeks later, and the LN fragments and dLNs were excised en bloc for histological examination.

**FIGURE 2:**
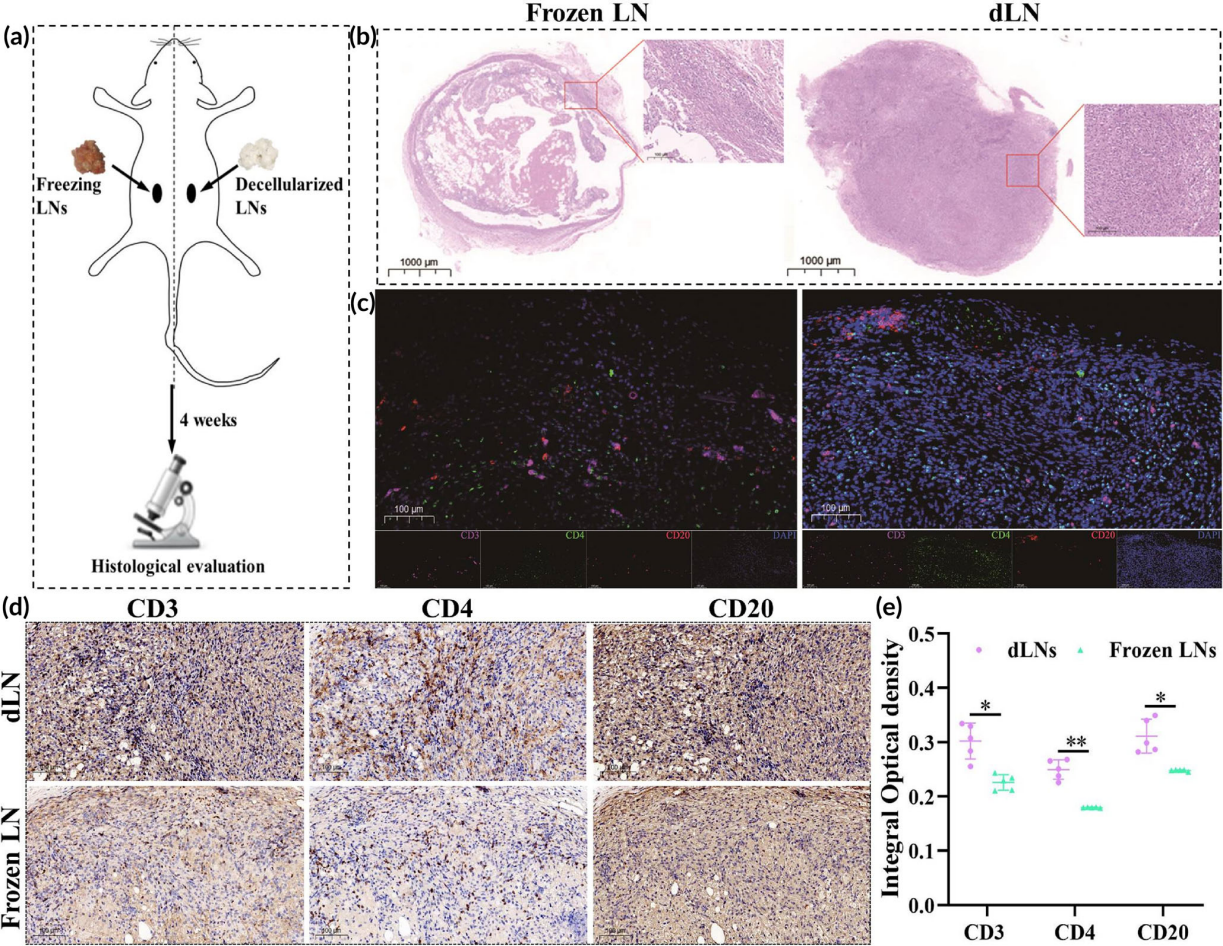
Decellularized lymph nodes can be used to induce the infiltration of immune cells in vivo. (a) Diagram of the dLNs transplantation into the back of mice. (b) HE staining of frozen LNs (left) and dLNs (right) transplanted into the backs of mice for 4 weeks. Scale bar = 1000 μm or 100 μm. (c) The multiplex immunofluorescence results of CD20, CD4, and CD3 for frozen LNs (left) and dLNs (right) transplanted into the backs of mice for 4 weeks. Scale bar = 100 μm. (d) CD20, CD4, and CD3 immunohistochemistry results of dLNs (above) and frozen LNs (below) transplanted into the backs of mice for 4 weeks. Scale bar = 100 μm. (e) Comparison of integral optical densities of CD3, CD4, and CD20 in dLNs and frozen LNs (*n* = 5/group). * indicated *p* < 0.05, ** indicated *p* < 0.01. LNs, lymph nodes; dLNs, decellularized lymph nodes. The student's *t*‐test was used to compare dLNs to frozen LNs treated mice.

Thirty‐six SD rats were randomized into three groups in a 1:1 ratio using a random number table: Sham group, dLNs group, and control group. To facilitate the identification of LNs, 0.1 mL of 10% methylene blue dye (JUMPCAN, Tai Xing, China) was subcutaneously injected distally into the hindlimb. For the dLN group and the control group, according to a previous study,^31^ the inguinal skin was circumferentially incised, and the popliteal and inguinal LNs were excised along with the surrounding fat pad (Figure 2a). In the dLN group, 8 to 10 dLN fragments were transplanted into the rats and evenly distributed in the groin and popliteal fossa regions. In the sham group, only the inguinal skin was circumferentially incised, and the inguinal and popliteal LNs and their surrounding fat pads were preserved. All skin was intermittently sutured. The animals were subsequently sacrificed 2 and 4 weeks later (*n* = 18 each) (Figure 2), and the inguinal tissues and skin of the affected limbs were excised en bloc for histological examination. Treatment of animals and sample collection were not blinded as they were performed by one single investigator. Experimental measurements and analysis were blinded during the assessment and data analysis process, which was performed by two other investigators.

### 2.6 Hindlimb circumference

The knee circumferences were measured manually before the procedure and on postoperative days (PODs) 4, 8, 12, 16, 20, 24, and 28 (Figure 2d). The mean circumferences were obtained by taking the average of three measurements.

### 2.7 Indocyanine green lymphography

According to a previous study,^31^ a total of 0.1 mL of 1.25 mg/mL indocyanine green (ICG) (Dandong Yichuang Pharmaceutical Co., Ltd., Dan Dong, China) was subcutaneously, distally injected using a 30‐gauge needle in the affected limb preoperatively and immediately after surgery, and at the first, second, third, and fourth weeks after surgery (three samples/group). Subsequently, the movement of ICG from the injection site was traced by visualizing its fluorescence signal with a hand‐held ICG device (Shinevia, Chang Sha, China). Lymphography was performed again after the inguinal region was opened at the fourth week (three rats/group). The ICG fluorescence intensity was represented by the relative fluorescence units (RFU).^32^ RFU was analyzed using ImageJ software, and a higher RFU indicates more severe dermal backflow and edema.

### 2.8 Histological evaluation

The tissue was fixed in 4% paraformaldehyde solution at room temperature for more than 48 h, washed the tissue under running water, embedded in paraffin, sectioned using a rotary microtome, and cut into 4 μm‐thick sections. All sections were dewaxed with xylene and rehydrated with 100% to 75% ethanol before hematoxylin and eosin (H&E) staining, immunofluorescence staining, and immunohistochemical staining.

H&E staining was performed following the manufacturer's instructions with hematoxylin (5 min) and eosin solution (5 min) (Solarbio, China). After staining, the tissue sections were dehydrated with alcohol, cleared in xylene, and mounted in neutral resin. 4',6‐diamidino‐2‐phenylindole dihydrochloride (DAPI) (D9542, Sigma–Aldrich, St. Louis, USA) was also used to stain samples for up to 10 min to observe the cell nuclei.

Multiplex immunofluorescence (mIF) and immunohistochemistry were performed to visualize immune cells. Sections were stained for simultaneous visualization of three markers using the following antibodies: anti‐CD3 (1:200; HA720082, HuaBio, Ji Nan, China), anti‐CD20 (1:600; ab64088, Abcam, Cambridge, England), and anti‐CD4 (1:200; ER1706‐80, HuaBio, Ji Nan, China). At the beginning of each staining cycle, the sections were immersed in citrate buffer for antigen retrieval. The membranes were sequentially incubated with each of the three primary antibodies in a wet box at 4°C overnight. The second antibody was added to the tissue based on the corresponding species of the primary antibody. Finally, the sections were stained with DAPI for 10 min and mounted with mounting medium. Images were obtained using a Zeiss LSM 710 laser scanning confocal microscope (Zeiss, Oberkochen, Germany).

Immunofluorescence was performed as previously described.^33^ Briefly, an anti‐lymphatic vessel endothelial receptor‐1 (LYVE‐1; 1:100, HA210844, HuaBio, Ji Nan, China) antibody was used to detect lymphangiogenesis. Secondary antibodies were conjugated to goat anti‐rabbit horseradish peroxidase or donkey anti‐rabbit‐488 (A32790, Thermo Fisher Scientific, Waltham, USA). DAPI was used for nuclear counterstaining. LYVE‐1‐positive LVs were counted manually in three microscopic fields in each stained sample. Three sections of each rat were counted, and the mean value was used for statistical analyses. Ultimately, the Image J software was used to count the number of LYVE‐1^+^ LVs and analyze the integrated optical density (IOD) of immunohistochemistry.

### 2.9 Statistical analysis

Normally distributed data are represented by the means ± standard deviations (SDs), while data with a skewed distribution are represented by the medians. A two‐tailed student's *t*‐test was used for comparisons between two groups, and one‐way analysis of variance (ANOVA) with the least significant difference (LSD) was used for comparisons among more than two groups. The Mann–Whitney *U* test was used to compare the number of lymph nodes or dLNs that were interconnected with LVs. The Fisher exact probability test was used for the analysis of categorical data. Statistical analysis was performed using SPSS 29.0 software (IBM Corp., Armonk, NY, USA, Version 29.0) and GraphPad Prism 8 software (GraphPad Software). Power analysis was performed using GPower 3.1.9.7 (Universitat Kiel, Germany). The observed power values, the statistical test performed, and the obtained *p*‐value are presented in Table S1, Supporting Information. *p* < 0.05 was considered to indicate statistical significance.

## 3 RESULTS

### 3.1 Preparation and characterization of porcine dLNs

Porcine dLNs were successfully prepared using SDS and Triton X‐100 (Figure 1a). HE (Figure 1b) and DAPI staining (Figure 1c) confirmed the absence of visible cells and nuclei, indicating that almost no cellular remnants were present. SEM revealed that dLNs had a spatial structure similar to that of normal LNs (Figure 1d). The DNA content of dLNs was much lower than that of normal LNs (0.017 ± 0.0005 vs. 1.399 ± 0.048 μg/mg, *p* < 0.05) (Figure 1e). Four weeks after the transplantation of frozen LNs and dLNs into mice (Figure 2a), frozen LNs formed obvious fibrous capsules, but dLNs did not (Figure 2b), indicating that dLNs have potential advantages in biocompatibility. Compared to frozen LNs, dLNs better induced CD20^+^, CD4^+^, and CD3^+^ immune cells repopulation (Figure 2c,d). As shown in Figure 2e, compared with frozen LNs, the IOD of CD3 (0.302 ± 0.033 vs. 0.226 ± 0.014, *p* < 0.05), CD4 (0.250 ± 0.018 vs. 0.179 ± 0.001, *p* < 0.01) and CD20 (0.312 ± 0.031 vs. 0.248 ± 0.001, *p* < 0.05) in dLNs was higher, indicating that dLNs had higher infiltration of T cells and B cells.

### 3.2 dLNs alleviate rat hindlimb edema

**FIGURE 3:**
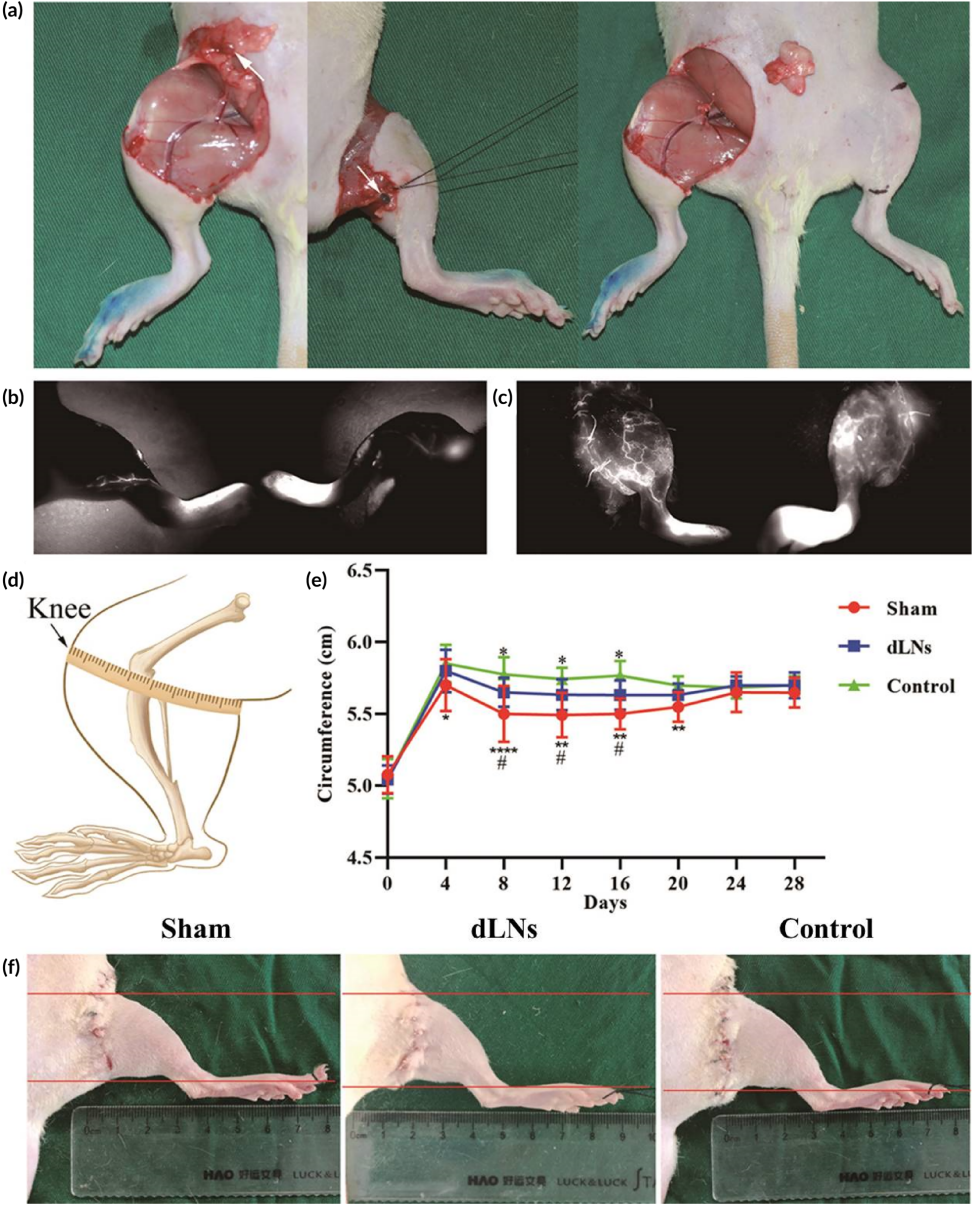
Inguinal and popliteal lymphatic dissection and changes in hindlimb circumference. (a) Lymphatic dissection. Methylene blue was used to visualize the inguinal and popliteal LNs (left and middle, white arrows), and the popliteal and inguinal LNs were excised along with the surrounding fat pads (right). (b) ICG lymphography results before modeling. (c) ICG lymphography immediately after lymphatic dissection. (d) Schematic diagram for measuring the hindlimb knee circumferences of the rats. (e) Differences in knee circumferences between mice in different groups (sham, dLN, and control) after surgery over the course of 4 weeks (* indicated the control group vs. the sham group, *p* < 0.05; **indicated the control group vs. the sham group, *p* < 0.01; *** indicated the control group vs. the sham group, *p* < 0.0001; # indicated the dLN group vs. the sham group, *p* < 0.05; * indicated the dLN group vs. the sham group, *p* < 0.05; before the second week after surgery, *n* = 12/group; after the second week after surgery, *n* = 6/group). The ANOVA with the least significant difference was used to compare the three groups. (f) Typical images of the affected hindlimbs on POD 12. LNs, lymph nodes; ICG, indocyanine green; PODs, postoperative days.

To explore the effectiveness of dLNs in the prevention of lymphedema, popliteal and inguinal LNs were excised along with the surrounding fat pads in the dLN group and the control group (Figure 3a). Preoperative ICG lymphography revealed intact lymphatic systems in the hindlimbs (Figure 3b). However, after the lymphatic systems were damaged to interrupt lymphatic drainage, LV dilation and dermal backflow were immediately observed (Figure 3c). To assess oedema, the circumferences of the affected limbs were measured; the control group had a significantly greater average circumference than the sham group on PODs 4, 8, 12, 16, and 20, and the dLN group had a significantly greater average circumference than the sham group on PODs 8, 12, and 16. The control group had a greater average circumference than the dLN group on PODs 8, 12, and 16 (Figure 3e and Table 1), indicating that oedema resolved more quickly in the dLN group and the sham group. On POD 12, the control group had the most severe limb swelling, followed by the dLN group, and the sham group had the mildest oedema (Figure 3f).

**TABLE 1:** Comparison of hindlimb circumferences in rats (cm).

Group	Pre (*N* = 36)	PODs 4 (*N* = 36)	PODs 8 (*N* = 36)	PODs 12 (*N* = 36)	PODs 16 (*N* = 18)	PODs 20 (*N* = 18)	PODs 24 (*N* = 18)	PODs 28 (*N* = 18)
Sham	5.1 ± 0.1	5.7 ± 0.2	5.5 ± 0.2	5.5 ± 0.2	5.5 ± 0.1	5.6 ± 0.1	5.7 ± 0.1	5.7 ± 0.1
dLNs	5.0 ± 0.1	5.8 ± 0.2	5.7 ± 0.1	5.6 ± 0.1	5.6 ± 0.1	5.6 ± 0.1	5.7 ± 0.1	5.7 ± 0.1
Control	5.1 ± 0.1	5.9 ± 0.1	5.8 ± 0.1	5.7 ± 0.1	5.8 ± 0.1	5.7 ± 0.1	5.7 ± 0.1	5.7 ± 0.1
P_0_	0.623	0.024	0.000	0.008	0.008	0.008	0.564	0.534
P_1_	0.512	0.123	0.016	0.045	0.045	0.110	0.194	0.355
P_2_	0.870	0.434	0.042	0.045	0.045	0.194	0.772	0.755

*Note*: The one‐way ANOVA with the least significant difference was used to compare the three groups.

Abbreviations: Pre, preoperation; PODs, postoperative days; P_0_ indicated control group versus Sham group; P_1_ indicated dLNs group versus Sham group; P_2_ indicated control group versus dLNs group.

### 3.3 dLNs accelerate lymphatic drainage and decrease dermal backflow

**FIGURE 4:**
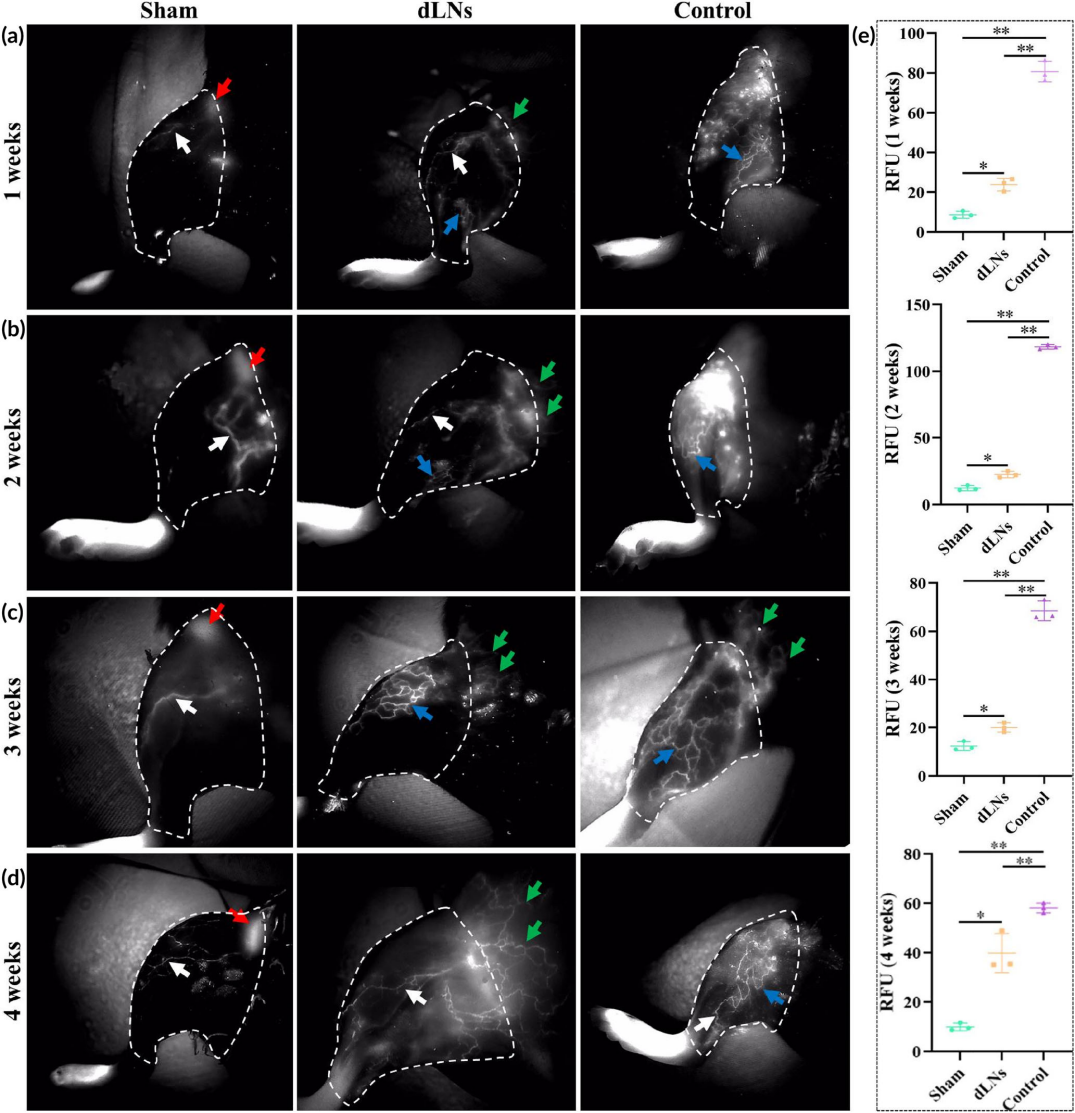
ICG lymphography results. (a) Typical ICG lymphography results at the first week after surgery. (b) Typical ICG lymphography results at the second week after surgery. (c) Typical ICG lymphography results at the third week after surgery. (d) Typical ICG lymphography results at 4 weeks after surgery. (e) The above data showed the relative fluorescence intensity analysis of the region of interest (the area with white dotted lines) from week 1 to week 4 at (*n* = 3/group). Red arrow: lymph node; white arrow: linear lymphatic vessels; green arrow: neonatal lymphatic vessels from the ventral side; blue arrow: flexed and dilated lymphatic vessels. * indicated *p* < 0.05, ** indicated *p* < 0.01. dLNs: decellularized lymph nodes; RFU: relative fluorescence unit; ICG, indocyanine green. The ANOVA with the least significant difference was used to compare the three groups.

To explore the impact of dLNs on lymphatic drainage, lymphography was used to detect dermal backflow and the presence of LNs. The sham group exhibited intact lymphatic drainage with linear LVs (white arrow) and LNs (red arrow) in the hindlimbs. The pattern of lymphatic drainage in the dLNs group was similar to that in the sham group, but with a few flexed and dilated LVs (blue arrows) from the first to third week. In contrast, in the control group, there were obvious flexed and dilated LVs (blue arrows) from the first to fourth week, with linear LVs (white arrow) only appearing in the distal hindlimbs by the fourth week (Figure 4a–d). Moreover, the dLNs group had ventral LVs connected to the inguinal region from the first to fourth week. The control group only showed this connection in the third week (green arrow). From the first to fourth week, the control group RFU was significantly higher than that of the dLNs and sham groups, indicating remarkable dermal backflow. Additionally, the dLNs group RFU was higher than the sham group (Figure 4e). These findings are consistent with the changes in volume (Figure 3e).

### 3.4 dLNs induce immune cell infiltration and integrate into the preexisting lymphatic system

**FIGURE 5:**
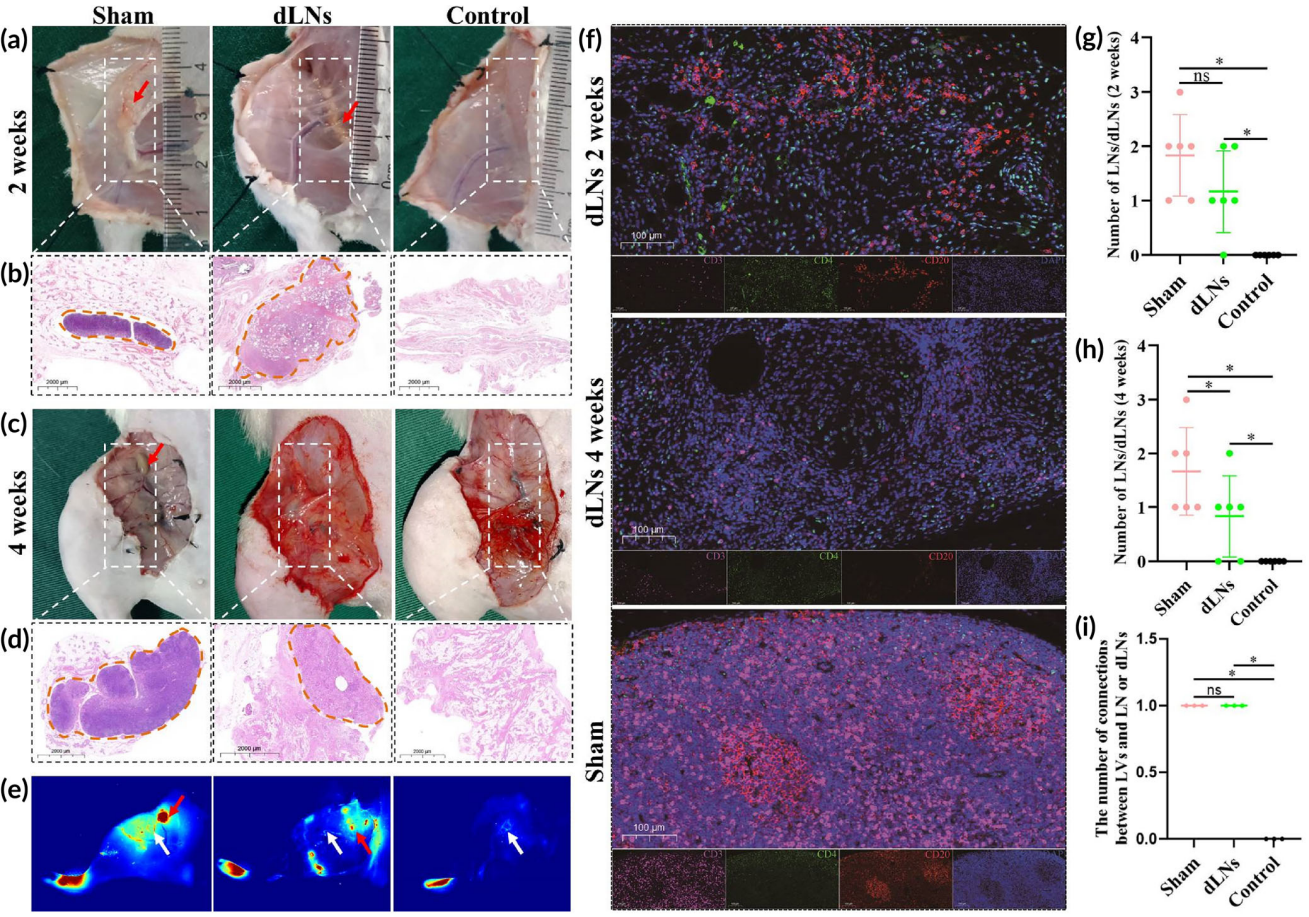
dLNs induce immune cells infiltration and integrate into the preexisting lymphatic system. (a) Real‐time image after the inguinal region was opened at the second week, the red arrows point to the LNs or transplanted dLNs. (b) The H&E staining results of the inguinal tissue at the second week. The red box indicates the location of LNs or the transplanted dLNs. Scale bar = 2000 μm. (c) Real‐time images after the inguinal region was opened at the fourth week. (d) The H&E staining results of the inguinal tissue at the fourth week. Scale bar = 2000 μm. (e) LNs or transplanted dLNs (red arrow) and LVs (white arrow) were revealed by ICG lymphography (pseudocolour pattern) after opening the inguinal region at the fourth week. (f) CD20, CD4, and CD3 mIF results for inguinal tissues from the sham group and dLN group at the second week and the fourth week after surgery. Scale bar = 100 μm. (g) The count of LNs or transplanted dLNs under H&E staining at the second week after surgery (*n* = 6/group). (h) The count of LNs or transplanted dLNs under H&E staining at the fourth week after surgery (*n* = 6/group). (i) The number of connections between LVs and LN or dLNs in rats (*n* = 3/group) (*p* = 0.018). * indicated *p* < 0.05. The ANOVA with the least significant difference was used to compare the number of LN or dLNs. The Mann–Whitney *U* test was used to compare the number of lymph nodes or dLNs that were interconnected with LVs. dLNs, decellularized lymph nodes; LNs, lymph nodes; H&E, hematoxylin‐eosin; ICG, indocyanine green; mIF, multiplex immunofluorescence; LVs, lymphatic vessels.

To validate the lymphography findings, HE staining and mIF were performed postoperatively at the second week and fourth week. The sham group exhibited typical LNs at the second week (An average of 1.83 ± 0.75 LNs per rat) (Figure 5a,b,g) and fourth week (An average of 1.67 ± 0.82 LNs per rat) (Figure 5c,d,h). The dLN group exhibited transplanted dLNs at the 2nd week (an average of 1.17 ± 0.75 LNs per rat) (Figure 5a,b,g), and no dLNs or typical LNs were observed at the fourth week (Figure 5c); however, HE staining revealed transplanted dLNs (an average of 0.83 ± 0.75 LNs per rat) (Figure 5d,h). These findings indicated that the transplanted dLNs were gradually degraded or absorbed. Moreover, the control group showed fragmented tissue (Figure 5a–d,g,h).

At 4 weeks postsurgery, when the groin area was opened, typical LNs (red arrow) and connected LVs (white arrow) were observed in the sham group by lymphography (Figure 5e). The dLN group exhibited a cluster of high fluorescence intensity (red arrow) and connected LVs (white arrow). Inversely, the control group showed only linear LVs (white arrow), and there were significant differences between the dLNs group and the control group (Figure 5e). This phenomenon was observed in all rats that underwent ICG angiography in the sham group (*n* = 3) and the dLNs group (*n* = 3) (Figure 5i). In the second week and fourth week, mIF confirmed that dLNs induced immune cells in the inguinal region, and the infiltrating cell type was similar to that of LNs (Figure 5f), suggesting that transplanted dLNs induced the infiltration of T cells and B cells and integrated into the preexisting lymphatic system. However, by the fourth week (Figure 5f), the number of infiltrating immune cells had decreased significantly, yet the pattern of cell distribution remained similar. This suggests that the transplanted dLNs may have been degraded, consistent with the ICG lymphangiography findings.

### 3.5 dLNs alleviate oedema by inducing LVs regeneration

**FIGURE 6:**
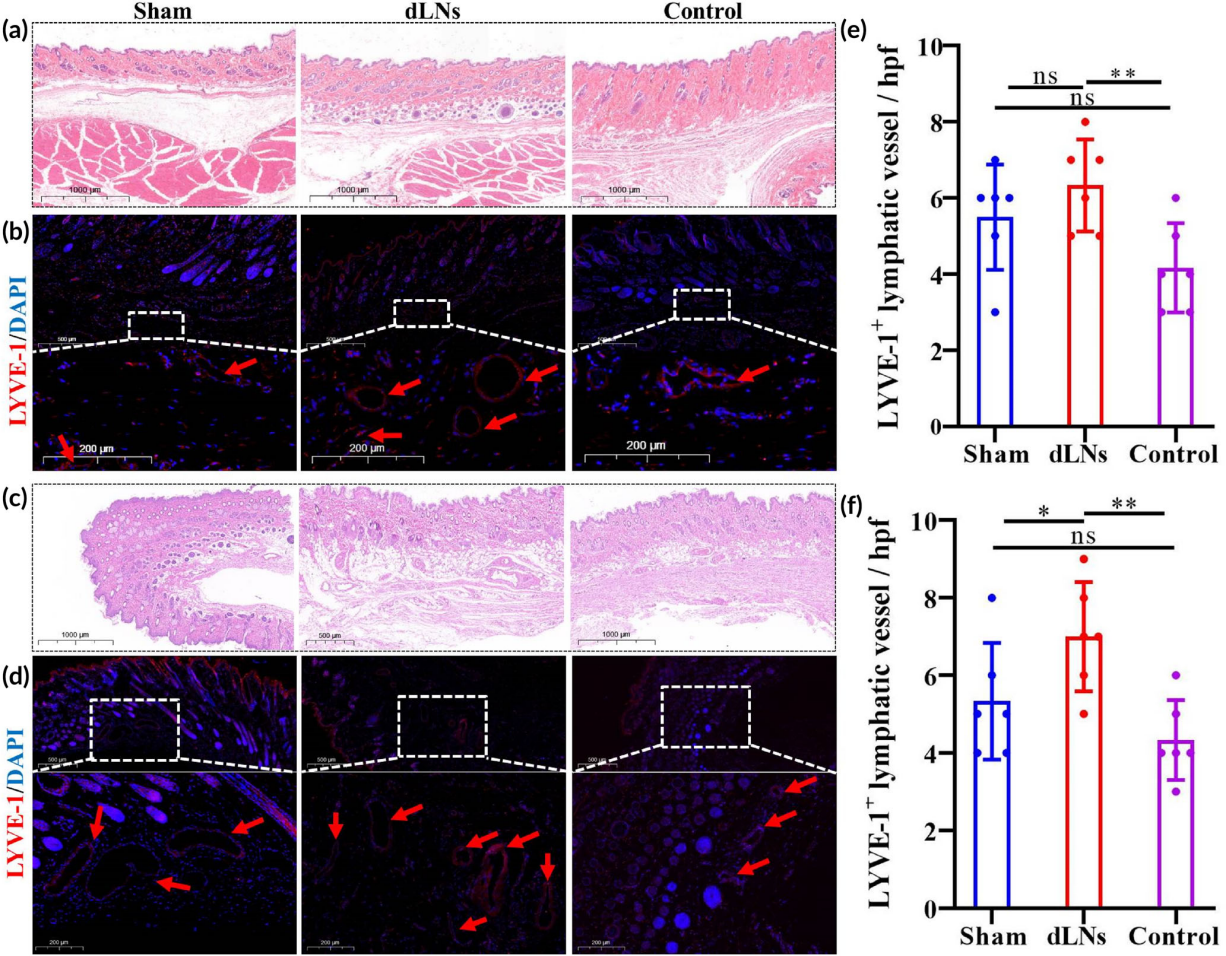
dLNs induce LV regeneration. (a) H&E staining results of the affected limb skin at 2 weeks after surgery. Scale bar = 1000 μm. (b) LYVE‐1 immunofluorescence results of the affected limb skin at the second week after surgery, and the red arrow points to LVs. Scale bar = 500 μm or 200 μm. (c) H&E staining results of the affected limb skin at 4 weeks after surgery. Scale bar = 1000 μm. (d) LYVE‐1 immunofluorescence results of the affected limb skin at 4 weeks after surgery, and the red arrow points to LVs. Scale bar = 500 or 200 μm. (e) Quantification of cutaneous LYVE‐1^+^ LVs at 2 weeks after surgery (** indicated *p* < 0.01, *n* = 6/group). (f) Quantification of cutaneous LYVE‐1^+^ LVs at 4 weeks after surgery (* indicated *p* < 0.05, ** indicated *p* < 0.01, *n* = 6/group). Red arrow indicated LVs. LVs, lymphatic vessels; H&E, hematoxylin‐eosin; LYVE‐1, lymphatic vessel endothelial receptor‐1. The ANOVA with the least significant difference was used to compare the three groups.

LVYE‐1 immunofluorescence was performed to examine LVs postoperatively at 2 and 4 weeks. HE staining revealed the skin structure in the three groups (Figure 6a,d). At 2 and 4 weeks postsurgery, the dLN group had more LYVE‐1^+^ LVs in the affected limb than did the control group (6.33 ± 1.21 vs. 4.17 ± 1.17, *p* = 0.009; 7.00 ± 1.41 vs. 4.33 ± 1.03, *p* = 0.003) (Figure 6e,f). There was no statistically significant difference between the dLN group and the sham group at the second week (6.33 ± 1.21 vs. 5.50 ± 1.38, *p* = 0.269) (Figure 6e), but the dLN group had more LVs than did the sham group at the fourth week (7.00 ± 1.41 vs. 5.33 ± 1.501, *p* = 0.047) (Figure 6f). There was also no statistically significant difference between the control group and the sham group at the second and fourth weeks (4.17 ± 1.17 vs. 5.50 ± 1.38, *p* = 0.086; 4.33 ± 1.03 vs. 5.33 ± 1.501, *p* = 0.214) (Figure 6e,f).

## 4 DISCUSSION

Lymphedema prevention remains challenging. Existing clinical techniques are largely limited in their clinical application due to factors such as patient compliance, the need for advanced professional skills, iatrogenic damage, and uncertain effectiveness.^6^, ^10^, ^11^ Therefore, novel prevention strategies need to be developed. Biomaterials have been used to prevent or treat lymphedema with positive effects, providing a new direction for preventing or treating lymphedema.^25^, ^34^, ^35^ Our results demonstrate that dLNs may provide a novel means for preventing lymphedema.

Injury to the lymphatic system is an initial factor for lymphedema, and restoring lymphatic drainage is crucial for reducing swelling.^8^, ^9^, ^36^ ILR and VLNT are two methods to prevent CRL by reconstructing lymphatic drainage. ILR involves directly draining lymph fluid into the venous system, whereas VLNT rebuilds lymphatic drainage through LN transplantation.^11^, ^12^ However, some studies have shown that ILR may increase the risk of cancer recurrence and metastasis^37^ and does not reduce the long‐term incidence of lymphedema.^37^, ^38^ VLNT has several drawbacks, such as the risk of donor site lymphedema, significant trauma, and limited donor sites, which restrict its clinical application.^14^, ^15^ Our findings showed the transplanted dLNs can induce the generation of LVs and the infiltration of immune cells. Thus, dLNs may replace VLNT or ILR, reducing patient trauma. Moreover, dLNs can induce T cells infiltration, which may have anti‐tumor potential. Studies have shown that skin CD4^+^ T cells infiltration is increased in CRL patients and is the main driver of lymphedema progression.^39^, ^40^ LNs have been proven to play a vital role in CD4^+^ T cells migration in lymphedema.^41^ Our results showed that dLNs transplantation induced CD4^+^ T cells infiltration, suggesting dLNs may draw CD4^+^ T cells from affected limbs to dLNs, thus preventing lymphedema progression. Moreover, strategies based on dLNs may offer a permanent alternative to traditional approaches (such as PSP and PSC) that require long‐term monitoring and depend on patient compliance. In the future, if confirmed in clinical studies, patients can be treated with day surgery or even on an outpatient basis. This could reduce hospital stays and associated healthcare costs.

Our results demonstrate good biocompatibility and show the potential of dLNs for inducing immune cell infiltration. Cuzzone et al.^26^ demonstrated that treating LNs with 0.075% SDS might be the most effective method for preparing dLNs. Lin et al.^27^ indicated that the preparation of dLNs with formic acid completely removed cellular components. However, the materials used were obtained from rat LNs, which greatly limits the widespread application of these dLNs. Choi et al.^28^ prepared porcine dLNs with 0.1% SDS, thereby expanding material sources. In our study, porcine dLNs were prepared using 1% SDS and 1% Triton X‐100. HE and DAPI staining confirmed a lack of cellular remnants (Figure 1), and the DNA content of the dLNs (<50 ng/mg dry tissue) met the standard for decellularized material.^42^ SEM revealed that the dLN extracellular matrix (ECM) maintained a three‐dimensional (3D) structure and micropores, which is beneficial for the infiltration and migration of immune cells (Figure 1d).^43^ Moreover, similar to previous studies,^26^, ^27^, ^28^ dLNs had good biocompatibility and could induce immune cell infiltration (Figure 2). However, dLNs were almost completely degraded and absorbed at the fourth week after transplantation, but there were still clusters of T cells and B cells in the transplantation area. Achieving the long‐term retention of dLNs in vivo will be one of the future research directions. However, a lack of stromal cells or lymphotoxin or lymphoid chemokines may be the main reason why the long‐term retention of dLNs does not occur in vivo.^17^, ^20^, ^43^

Engineered LN or aLN may be a solution. Suematsu et al.^20^ induced the formation of aLN in mice by injecting bovine collagen scaffolds and thymic stromal cells into the subcapsular space of the mouse kidney, which could trigger an immune response in severe combined immunodeficient mice. They summarized the following elements for preparing long‐term preserved aLNs: stromal cells that can replace lymphoid stromal cells, lymphotoxin or lymphoid chemokines, and biocompatible scaffolds to provide the 3D structures.^17^, ^20^, ^43^ However, current aLNs are often created using non‐LN derived materials as scaffolds, which cannot replicate the complex structure of LN.^16^, ^25^ dLNs might be a great scaffold for preparing aLNs. Lin et al.^27^ demonstrated that bone marrow derived dendritic cells can grow well on dLNs scaffolds and induce effective anti‐tumor immunity. In the hydrogel scaffold prepared with dLNs ECM, the LN microenvironment can be effectively simulated, and the function of macrophages can be regulated, thereby promoting the healing of injured tissues in vivo.^28^ Our results also indicated that dLNs show potential as scaffolds for preparing aLNs.

In this study, compared with those of the control group, the circumferences of the dLNs group were significantly reduced on PODs 8, 12, and 16, and those of the sham group were significantly reduced on PODs 4, 8, 12, 16, and 20 (Figure 2e). Postoperative lymphography could explain this phenomenon (Figure 3). The sham group had the fastest oedema recovery due to the presence of intact inguinal LNs and lymphatic drainage. In the dLN group, neonatal abdominal LVs were connected to hindlimb LVs from the first to fourth week postoperatively, but this connection was observed only until the third week in the control group, resulting in a faster recovery of oedema in the dLN group. However, as the lymphatic drainage recovered and the rats aged, the circumferences continued to increase and tended to be similar among the three groups. Lymphography also revealed that the fluorescence intensities in the inguinal region of the dLN group were similar to those of the sham group, but the fluorescence intensities of the control group increased significantly at the first and second weeks (Figure 3), suggesting that dLN transplantation enhanced lymphatic drainage and that backflow existed in the control group. Moreover, the number of fluorescence clusters in the inguinal regions of the mice in the dLN group decreased, and the LVs dilated at the third and fourth weeks, suggesting that LVs gradually decreased in size as dLNs were degraded and absorbed. Lenti et al.^21^ revealed endogenous LVs surrounding and connecting to aLNs by lymphography. At the fourth week, we again performed lymphography after the inguinal region was opened and observed a similar phenomenon (Figure 5e), indicating that dLNs were integrated into the existing lymphatic system.

Previous studies have shown that LNs act as transit stations for lymphatic drainage and secrete various lymphangiogenic factors to promote LVs regeneration.^44^, ^45^, ^46^ The infiltrating cells in transplanted dLNs may also promote LVs regeneration by secreting lymphangiogenic factors. Kang et al.^29^ reported that dLNs alone and dLNs combined with stem cells could increase the expression of vascular endothelial growth factor‐A. Lymphography revealed a dense network of LVs surrounding and connecting to transplanted dLNs (Figures 4 and 5e). These findings were confirmed by LYVE‐1 immunofluorescence and explained why oedema was alleviated more quickly in the dLN group. At 4 weeks postsurgery, although the number of LVs in all three groups increased, the number of LVs in the dLN group still significantly exceeded that in the sham group or the control group (Figure 6). Therefore, transplanted dLNs play an important role in preventing lymphedema, and as transplanted dLNs disappear, sole reliance on neonatal LVs may not be sufficient to counteract the progression of oedema.

Our study had several limitations. First, the progression of lymphedema is characterized by chronic inflammation dominated by infiltration of CD4^+^ T‐cells,^8^, ^9^, ^36^ further demonstration is needed to determine the origins of the infiltrating cells in dLNs and compare the differences in immune cell infiltration induced by different biomaterials. Moreover, porcine LNs from different batches may result in variations. We have begun to explore more stringent standardization procedures to overcome these differences and are also testing porcine pathogenology to ensure the safety of clinical applications. Second, we have not explored the detailed mechanism by which dLNs promote LV regeneration. Further detection of pro‐lymphangiogenic factors like vascular endothelial growth factor‐C and the expression of CD4^+^ T cell cytokines in the skin will provide more evidence. Finally, we found that dLNs can be retained only temporarily in vivo, and we are exploring making it into aLNs or boosting their hydrophobicity for long‐term in‐body retention and functionality.

## 5 CONCLUSIONS

In summary, this study provides preliminary preclinical evidence to confirm the effectiveness of porcine dLNs in preventing lymphedema. More importantly, our study showed that the transferred porcine dLNs can successfully promote LVs regeneration, which integrated into the preexisting lymphatic system to accelerate lymphatic drainage, thereby accelerating the recovery of edema. In addition, dLNs transplantation induced infiltration of CD4^+^, CD3^+^, and CD20^+^ immune cells, indicating its potential in restoring immune function. Although further studies are essential, this work suggests a new strategy for the prevention or treatment of lymphedema. These findings also provide evidence for dLNs as a scaffold for aLNs.

## AUTHOR CONTRIBUTIONS

ZW, CD, and XS conceived the original ideas of this manuscript, reviewed the manuscript, and executed supervision throughout the process. JY and JZ wrote the manuscript, carried out experiments, and analyzed the data. YJ, JZ, WP, YL, XL, YD, JC, CL, YZ, and YZ carried out experiments and data analysis. All authors have read and approved the manuscript.

## FUNDING INFORMATION

This work was supported by the Zunyi Medical University graduate research fund (ZYK155), the Talents Science Cooperation Platform of Guizhou ([2020]5012), Collaborative Innovation Center of Chinese Ministry of Education (2020‐39), the National Natural Science Foundation of China (82360445), Talents Science Platform of Zunyi City (No. 2021‐3), Shanghai Wang Zhengguo Trauma Medicine Development Foundation (SZYZ‐TR‐05), and the joint fund project of Zunyi City [(2023) 228]. The funding agents had no role in the study design, data collection, data analysis, interpretation, or report writing.

## CONFLICT OF INTEREST STATEMENT

None declared.

## ETHICS STATEMENT

All animal procedures were carried out in accordance with the principles of ARRIVE and approved by the Biomedical Research Ethics Committee of the Affiliated Hospital of Zunyi Medical University, Grant No. zyfy‐an‐2023‐0220.

## Supporting information


**Table S1.** The result of power analysis.

## Data Availability

The data that support the findings of this study are available on request from the corresponding author. The data are not publicly available due to privacy or ethical restrictions.
