# Supplementary material for: Decellularized lymph node scaffolds accelerate restoration of lymphatic drainage in rat hind limb lymphedema
Source: Bioeng Transl Med. 2025 Jul 31;10(6):e70056. doi: 10.1002/btm2.70056 (PMC12617546; doi:10.1002/btm2.70056)
Supplement: Supplementary file 1 — Table S1. The result of power analysis. [file BTM2-10-e70056-s001.docx]

Supplementary File

Supplementary Table1. The result of power analysis.

| Figure | Total sample | Type of test | Observed Power | p-value |
| --- | --- | --- | --- | --- |
| Figure 1e | 6 | student’s t-test | 1 | 0.0004 |
| Figure 2e CD3 | 10 | student’s *t*-test | 1.0 | <0.05 |
| Figure 2e CD4 | 10 | student’s *t*-test | 1.0 | <0.01 |
| Figure 2e CD20 | 10 | student’s *t*-test | 1.0 | <0.05 |
| Figure 3e Pre | 36 | one-way ANOVA with LSD | 0.84^a^ | 0.623 |
| Figure 3e Pre | 36 | one-way ANOVA with LSD | 1.0^b^ | 0.512 |
| Figure 3e Pre | 36 | one-way ANOVA with LSD | 1.0^c^ | 0.870 |
| Figure 3e PODs 4 | 36 | one-way ANOVA with LSD | 1.0 a | 0.024 |
| Figure 3e PODs 4 | 36 | one-way ANOVA with LSD | 0.86 ^b^ | 0.123 |
| Figure 3e PODs 4 | 36 | one-way ANOVA with LSD | 1.0 ^c^ | 0.434 |
| Figure 3e PODs 8 | 36 | one-way ANOVA with LSD | 1.0 ^a^ | 0.00006 |
| Figure 3e PODs 8 | 36 | one-way ANOVA with LSD | 1.0 ^b^ | 0.016 |
| Figure 3e PODs 8 | 36 | one-way ANOVA with LSD | 1.0 ^c^ | 0.042 |
| Figure 3e PODs 12 | 36 | one-way ANOVA with LSD | 1.0 ^a^ | 0.008 |
| Figure 3e PODs 12 | 36 | one-way ANOVA with LSD | 1.0 ^b^ | 0.045 |
| Figure 3e PODs 12 | 36 | one-way ANOVA with LSD | 1.0 ^c^ | 0.045 |
| Figure 3e PODs 16 | 18 | one-way ANOVA with LSD | 1.0 ^a^ | 0.008 |
| Figure 3e PODs 16 | 18 | one-way ANOVA with LSD | 0.93 ^b^ | 0.045 |
| Figure 3e PODs 16 | 18 | one-way ANOVA with LSD | 1.0 ^c^ | 0.045 |
| Figure 3e PODs 20 | 18 | one-way ANOVA with LSD | 1.0 ^a^ | 0.008 |
| Figure 3e PODs 20 | 18 | one-way ANOVA with LSD | 0.98 ^b^ | 0.110 |
| Figure 3e PODs 20 | 18 | one-way ANOVA with LSD | 1.0 ^c^ | 0.194 |
| Figure 3e PODs 24 | 18 | one-way ANOVA with LSD | 0.56 ^a^ | 0.564 |
| Figure 3e PODs 24 | 18 | one-way ANOVA with LSD | 0.19 ^b^ | 0.194 |
| Figure 3e PODs 24 | 18 | one-way ANOVA with LSD | 0.77 ^c^ | 0.772 |
| Figure 3e PODs 28 | 18 | one-way ANOVA with LSD | 0.53^a^ | 0.534 |
| Figure 3e PODs 28 | 18 | one-way ANOVA with LSD | 0.36^b^ | 0.355 |
| Figure 3e PODs 28 | 18 | one-way ANOVA with LSD | 0.76^c^ | 0.755 |
| Figure 4e (week 1) | 9 | one-way ANOVA with LSD | 1.0 ^a^ | 0.0000 |
| Figure 4e (week 1) | 9 | one-way ANOVA with LSD | 1.0 ^b^ | 0.0023 |
| Figure 4e (week 1) | 9 | one-way ANOVA with LSD | 1.0 ^c^ | 0.000001 |
| Figure 4e (week 2) | 9 | one-way ANOVA with LSD | 1.0 ^a^ | 0.0000 |
| Figure 4e (week 2) | 9 | one-way ANOVA with LSD | 1.0 ^b^ | 0.0008 |
| Figure 4e (week 2) | 9 | one-way ANOVA with LSD | 1.0 ^c^ | 0.0000 |
| Figure 4e (week 3) | 9 | one-way ANOVA with LSD | 1.0 ^a^ | 0.0000 |
| Figure 4e (week 3) | 9 | one-way ANOVA with LSD | 1.0 ^b^ | 0.016 |
| Figure 4e (week 3) | 9 | one-way ANOVA with LSD | 1.0 ^c^ | 0.0000 |
| Figure 4e (week 4) | 9 | one-way ANOVA with LSD | 1.0 ^a^ | 0.000018 |
| Figure 4e (week 4) | 9 | one-way ANOVA with LSD | 1.0 ^b^ | 0.000267 |
| Figure 4e (week 4) | 9 | one-way ANOVA with LSD | 1.0 ^c^ | 0.0035 |
| Figure 5g | 18 | one-way ANOVA with LSD | 1.0 ^a^ | 0.0001 |
| Figure 5g | 18 | one-way ANOVA with LSD | 0.91 ^b^ | 0.08 |
| Figure 5g | 18 | one-way ANOVA with LSD | 0.99 ^c^ | 0.005 |
| Figure 5h | 18 | one-way ANOVA with LSD | 0.99 ^a^ | 0.0004 |
| Figure 5h | 18 | one-way ANOVA with LSD | 0.97 ^b^ | 0.04 |
| Figure 5h | 18 | one-way ANOVA with LSD | 1.0 ^c^ | 0.04 |
| Figure 5i | 9 | Mann-Whitney *U* test | 0.51 | 0.018 |
| Figure 6e | 18 | one-way ANOVA with LSD | 0.99 ^a^ | 0.086 |
| Figure 6e | 18 | one-way ANOVA with LSD | 0.92 ^b^ | 0.269 |
| Figure 6e | 18 | one-way ANOVA with LSD | 1.0 ^c^ | 0.009 |
| Figure 6f | 18 | one-way ANOVA with LSD | 0.99 ^a^ | 0.214 |
| Figure 6f | 18 | one-way ANOVA with LSD | 0.98 ^b^ | 0.047 |
| Figure 6f | 18 | one-way ANOVA with LSD | 1.0 ^c^ | 0.003 |

LSD, least significant difference; ^a^ indicated control group vs. Sham group; ^b^ indicated dLNs group vs. Sham group; ^c^ indicated control group vs. dLNs group.
